# Influential Effects of Emotional Intelligence on the Relationship between Job Stress and Burnout among General Hospital Administrative Staff

**DOI:** 10.3390/healthcare10020194

**Published:** 2022-01-20

**Authors:** Woosok Han, Jinkyung Kim, Junghee Park, Mihyang Lee

**Affiliations:** 1 Department of Hospital Management, Konyang University, Daejeon 35365, Korea; wshan@konyang.ac.kr; 2 Department of Emergency Medical Service, Konyang University, Daejeon 35365, Korea; jhpug@konyang.ac.kr; 3 Department of Nursing, Konyang University, Daejeon 35365, Korea; haha@konyang.ac.kr

**Keywords:** job stress, burnout, emotional intelligence, hospital administrative staff

## Abstract

Emotional intelligence plays an important role at the time of determination of job stress or in controlling emotions arising from job stress. This study uses a cross-sectional descriptive design to assess the extent of job stress, emotional intelligence, and burnout of general hospital administrative staff, and to identify an influencing effect of emotional intelligence on the relationship between job stress and burnout. Data were collected by using a structuralized questionnaire survey conducted on 191 administrative staff at 4 general hospitals in a metropolitan city in Korea in September 2021. The effects of emotional intelligence on the relationship between job stress and burnout were analyzed by using hierarchical multiple regression analysis. The results of analysis showed: (a) job stress and burnout displayed positive correlation (r = 0.57, *p* < 0.001) while (b) emotional intelligence and burnout displayed negative correlation (r = −0.26, *p* < 0.001), and (c) factors with significant effects on burnout included age (≥40 years), job stress, and emotional intelligence. Emotional intelligence had direct (independent) effects on burnout. Thus, the improvement of emotional intelligence is necessary to decrease burnout levels in general hospital administrative staff.

## 1. Introduction

General hospital administrative staff play the role of providing and processing various data necessary for managerial decision making by effectively operating manpower, facilities and budgets through coordination and management of conflicting interests among various departments, such as medical treatment, nursing, and medical support departments [1,2]. In particular, general affairs, personnel management, planning, and accounting are the departments that play central roles in the hospital in charge of vision and performances while ensuring that the hospital is being operated harmoniously at the same time by planning, executing, and inspecting core issues of hospital management. As such, the roles of administrative staff working in such departments are being emphasized [3].

Healthcare professionals working at medical institutions are more sensitive to job stresses due to abrupt medical errors related to patients, chronic tension, conflicts in interpersonal relationships, overwork, ambiguity in the roles given, and irrational and authoritative culture in the workplace experienced in the process of conducting their respective duties [4]. In particular, since administrative staff need to engage in interaction with people of diverse occupations, including physicians, nurses, and medical technicians, in the process of delivering diversified and complicated medical services to patients within limited time, conflicts would be induced, thereby aggravating job stress [5]. In addition, job stress can increase due to stress from conflict with senior staff over work instructions and priority within the organization, and roles with a lack of necessary work hours and excessive workload [6]. Burnout of general hospital administrative staff occurs in the work environment when a high level of job stress is experienced repetitively [2,7]. Burnout refers to the physical and mental exhaustion syndrome that leads to loss of enthusiasm, tiredness without reason, and negative attitudes towards the job [8]. Burnout is induced by overwork, the feeling that reality is unfair, loss of meaning of work, and conflict with fellow workers; job stress of administrative staff can further lead to degraded productivity, as well as inadequate quality of medical services [1,7]. 

Bakker and Demerouti introduced the job demands-resources (JD-R) theory that suggests all occupations have work-related job stress, and to that end each job has different factors that affect burnout [9]. Studies show that hospital administrative staff experience a high level of job stress due to the possibility of medical accidents occurring during treatments, conflicts in interpersonal relationships with physicians and nurses in the process of work, and a lack of autonomy in work. [1,5]. Literature on job stress and burnout among general hospital administrative staff argues that administrative staff suffer extensive job stress due to the excessiveness of roles, including overwork and role conflicts, thereby experiencing burnout [2,6]. 

Emotional intelligence refers to the ability to protect oneself from irrational thoughts that become the cause of psychological oppression and plays important role in controlling emotion manifested due to job stress [10,11]. Emotional intelligence can enhance satisfaction with hospital services by enhancing work performance and efficiency by enabling one to concentrate on fair and stable work execution through sympathizing or understanding of the emotions of patients, as well as fellow workers including physicians and nurses [11,12,13,14]. Emotional intelligence of administrative staff is an element that needs to be focused on intensively in order to improve performance and competitiveness of hospitals. A majority of studies are on the emotional intelligence of nurses, and their relationship between job stress and burnout [11,12,13,14]; however, there is a marked lack of studies with general hospital administrative staff as study participants [15]. Kim and Bae [15] assessed that the emotional intelligence of general hospital administrative staff increases with the increases in ego resilience, loyalty to the organization, and dedication to the organization. Since emotional intelligence plays an important role in determining the presence of job stress and in controlling emotion that demonstrates stress, it is important to assess them among general hospital administrative staff. Therefore, this study assessed the extent of job stress, emotional intelligence, and burnout among general hospital administrative staff, and analyzed the effects of emotional intelligence on the relationship between job stress and burnout. Moreover, the study results would provide basic data to design intervention strategies aimed at reducing the burnout of general hospital administrative staff.

## 2. Materials and Methods

### 2.1. Study Design

This is a cross-sectional descriptive study to examine the effects of the job stress of general hospital administrative staff on burnout, and to verify the effects of emotional intelligence on the relationship between job stress and burnout.

### 2.2. Sample

Using G*Power (version 3.1.9.7., Heinrich-Heine-Universität Düsseldorf, Düsseldorf, Germany) program, the sample size calculated with a significance level(α) of 0.05, a medium effect size of 0.15, statistical power of 0.90, and 11 independent variables in multiple regression method was 172 participants. The inclusion criteria are: administrative staff who have worked in general hospitals for more than six months and have agreed to participate in the study. The exclusion criteria were: workers with less than six months work experience and either contract or outsourced workers. Considering a 15% dropout rate, a total of 200 administrative staff participated in the study. After excluding 9 due to incomplete surveys, 191 participants were included for the analysis. 

### 2.3. Measures

A structuralized questionnaire was used for this study. The contents of the questionnaires were composed of 8 questions on study participants’ general characteristics, 24 questions on job stress, 14 questions on emotional intelligence, and 9 questions on burnout, for a total of 55 questions.

#### 2.3.1. Job Stress

We used the Korean Occupational Stress Scale Short Form (KOSS-SF) developed by Chang et al. [16] to measure job stress. The measure consists of 21 questions in 7 domains, rated on a 4-point Likert scale ranging from 1, “not at all”, to 4, “very much”. A higher score implies a more extensive experience of job stress. For assessing the overall level of job stress, the scores for each domain were converted into a 100 points scale, which were then summed before being divided by 7 again [16]. The reliability of job stress scale in this study was Cronbach’s α = 0.83, which was similar to 0.82 in the Chang et al. study [16]. 

#### 2.3.2. Emotional Intelligence 

Emotional intelligence was measured by the Wong and Law Emotional Intelligence Scale (WLEIS), developed by Wong and Law [10]. It consists of a total of 14 questions (7-point Likert scale). Higher scores signify higher emotional intelligence. The reliability of the emotional intelligence scale was Cronbach’s α = 0.87, when the reliability scores range from 0.76 to 0.89 in the original study [10]. 

#### 2.3.3. Burnout

We used the Maslach Burnout Inventory (MBI) developed by Maslach [8], converted into Korean [11]. Burnout was measured with the nine questions on emotional burnout sub-domain [17], rated on a 5-point Likert scale ranging from 1, “not at all”, to 5, “very much”. The higher the score, the higher the emotional burnout. The reliability of emotional burnout scale was Cronbach’s α = 0.89, compared to 0.94 in the Song’s study [17].

### 2.4. Data Collection

This study was approved by the Institutional Review Board (IRB) of Konyang University (KYU-2021-06-019). Data were collected from 17 September to 30 September 2021. For data collection, approval from the study hospitals were acquired after having explained the purpose and details of the study to the head of the corresponding administrative departments in each general hospital. Four general hospitals situated in Daejoen Metropolitan City, South Korea participated in the study. An online survey was conducted using a Google Form. In order to control duplicate responses, study participants were required to enter personal cell phone numbers, which were cross-checked with sociodemographic characteristics. Study participants were asked to voluntarily participate in the survey and gave online consent through the survey questionnaire URL posted in each medical institutions’ intranet. 

### 2.5. Data Analysis

Data were analyzed with SPSS 21.0 (IBM Corp., Armonk, NY, USA) program. Frequency and descriptive statistics were executed for general characteristics of study participants and independent variables. Differences in burnout on individual factors were analyzed using Chi-square, independent *t*-test, and ANOVA. The Scheffe test was performed for the post hoc analysis. Pearson’s correlation coefficients were computed to confirm the correlation among job stress, emotional intelligence, and burnout. The statistical significance level was set at α = 0.05. Shapiro-Wilk statistics confirmed the normality of data (*p* = 0.95, α = 0.05). Hierarchical multiple regression analysis was performed to identify factors that influence burnout and to verify the effects of emotional intelligence on the relationship between job stress and burnout. The problems of multicollinearity were checked. In Model 1, the general characteristics of study participants were considered. To determine and compare the effects of factors on burnout, Model 2 further included job stress and emotional intelligence, and, in Model 3, the interaction effect between job stress and emotional intelligence was added.

## 3. Results

### 3.1. Differences in Burnout by General Characteristics

Table 1 shows the differences in burnout by general characteristics. There was a total of 191 participants with female, married, university graduates, and those without religion accounting for 61.26%, 52.88%, 67.02%, and 58.64% of the participants, respectively. The average age was 35.7 years and the average length of career was 10.1 years. There were 116 (60.7%) participants who worked in the departments with no contact with patients and 155 (81.1%) were team members. The statistically significant difference in burnout was observed by gender (t = −1.9, *p* < 0.10). Females showed higher extent of burnout compared to males.

### 3.2. Properties of Job Stress, Emotional Intelligence and Burnout Questionnaries

Mean scores for job stress, emotional intelligence, and burnout of participants were 47.5 out of 100, and 5.28 and 2.92 out of 7, respectively (Table 2). 

### 3.3. Correlations among Job Stress, Emotional Intelligence and Burnout of Subjects

Correlations among job stress, emotional intelligence, and burnout of the subjects are shown in Table 3. Burnout showed a significant positive correlation with job stress (r = 0.56, *p* < 0.001, R^2^ = 0.31) and a significant negative correlation with emotional intelligence (r = −0.26, *p* < 0.001, R^2^ = 0.07), while emotional intelligence had a significant negative correlation with job stress (r = −0.26, *p* < 0.001, R^2^ = 0.07).

### 3.4. Factors Influencing Burnout

Table 4 presents the results of hierarchical multiple regression analysis. Model 1 includes gender, age, marital status, religion, and affiliated departments; in Model 2, job stress and emotional intelligence are added. Model 3 includes the interaction effect between job stress and emotional intelligence to verify whether emotional intelligence has a mediating effect. The effects of variables were analyzed with the explanatory power (R2) and increased portion of the revised explanatory power (Adj. R2) within the significance level (*p* < 0.001) as the basis. The Model 2 and 3 were statistically significant (*p* < 0.001). In Model 2 and Model 3, the variation inflation factor (VIF) were in the range of 1.0~2.8, which was well within the threshold of 10, there was no problem of multicollinearity. 

In Model 2, factors that affect burnout were age over 40 years, job stress, and emotional intelligence. These variables explained burnout with explanatory power of 35.4% (F = 14.00, *p* < 0.001). Job stress (β = 1.09, *p* < 0.001) was a significant variable that imparts positive effects on burnout, while age over 40 years (β = −0.39, *p* < 0.05) and emotional intelligence (β = −0.14, *p* < 0.10) had significant negative effects on burnout. Model 3, for which job stress, emotional intelligence, and interaction between job stress and emotional intelligence have been inputted, displayed explanatory power of 38.2%, which is an increase of 0.1% compared to Model 2, with no mediating effects of emotional intelligence. Emotional intelligence was found to have an independent and direct negative effect on burnout.

## 4. Discussion

This study attempted to understand the relationship between job stress and burnout in administrative staff. According to the job demand–resource (JD-R) theory [9], work characteristics are associated with job stress. The study could provide insight on the degree of job stress for administrative staffs working in general hospitals and the effect of emotional intelligence, at an individual level, on job stress and burnout.

The participants’ average job stress score was 47.5, which was similar to the score of 47 in the Kim and Bae [15] study conducted on general hospital administrative staff. This score was higher than administrative staffs in all types of medical institutions at 38.7 [1] and various healthcare professionals (including nurses, medical technicians, and administrative staff) in general hospitals at 45.7 [18]. This illustrates that the extent of job stress could differ depending on the types of medical institutions or occupations that the study participants are affiliated with. Previous studies on job stress in administrative staff show that a higher level of stress is strongly associated with longer work hours, low level of social support, and workload [19,20]. Since administrative staff are core personnel who interact with constituent members and support medical services in healthcare organizations, it is critical to pay attention and interest to human resources management by assessing the extent of their job stresses in consideration of the quality of treatment for patients [1,21]. Therefore, there is a need for a study that analyzes the causes of job stress experienced by general hospital administrative staff and develops intervention strategies accordingly.

The score for emotional intelligence was 5.29 out of 7, which can be converted into 75.57 out of 100, which is similar to the score of 74.85 in the Kim and Bae [15] study. Since emotional intelligence of general hospital administrative staff enables them to sympathize with and understand the emotions of patients, guardians, and fellow workers, and to concentrate on the fair execution of work, it is necessary to improve the emotional intelligence of administrative staff. Studies [9,20] have shown that emotional intelligence of general hospital administrative staff is related to gender, smoking, and health related activities. By developing emotional intelligence capabilities, administrative staff could enhance the social network in healthcare organizations [22]. Additional research is needed to understand the extent of their emotional intelligence and to verify factors that affect their emotional intelligence.

The score for emotional burnout of general hospital administrative staffs was 2.92 out of 5, which can be converted into 58.4 out of 100. This was lower than the score of 62.0 for general hospital administrative staff in Lim’s study [23]. Maslach and Jackson [24] presented emotional burnout, personal sense of accomplishment, and depersonalization as the subordinate factors of burnout. The study result was different from those of the preceding study since only emotional burnout was analyzed in this study. When the extent of burnout is observed in accordance with the general characteristics, there was no significant difference in terms of gender, experience, marital status, educational level, religion, position, and affiliated department. However, Ziaei et al. [20], targeting administrative staff, showed differences in age, work experience, gender, and educational level. The age effect showed that emotional burnout is lower for those in their forties or older in comparison to that for those in their twenties. Although Lim’s study [23] showed similar results with no significant difference in terms of educational level, position, and work experiences, the effects of age were different. The age effect on burnout was also reported in previous studies, conducted on nurses [12,13,25] and on administrative staffs in hospitals [20,26], and coincided with the results of this study. The level of emotional burnout in administrative staff was associated with work-related dimensions and was similar to that of other types of healthcare professionals [21,27]. Although most studies focus on burnout among medical personnel, research on administrative staff is relatively insufficient. Further research is recommended to explore the factors affecting burnout in administrative staff. 

Emotional burnout increased with an increase in job stress and a decrease in emotional intelligence among general hospital administrative staffs, and emotional intelligence decreased with an increase in job stress. The results are similar to those of the preceding studies that job stress increases burnout [2,27] and emotional intelligence imparts negative effects on burnout [11,12,13,14]. Burnout appeared as a response to job stress, and job stress management in administrative staff is an important factor in lowering the level of burnout. It was confirmed in other studies [28,29] that higher emotional self-awareness and emotional regulation were associated with a lower level of burnout. In order to reduce the exhaustion of administrative staff, it is necessary to develop an intervention strategy that can reduce job stress and increase emotional intelligence. 

In the relationship between job stress and burnout, emotional intelligence had independent and direct effects on burnout, which was similar to the results of preceding study that analyzed direct [12] or mediating [13,30] effects of emotional intelligence. Administrative staff interact with patients, guardians, and other healthcare professionals, and experience emotional inconsistency because they suppress their emotions and perform their duties to follow hospital rules. This inconsistency of emotions leads to stress which would eventually lead to burnout from dissatisfaction with their job [21]. Some studies on burnout in nurses illustrated emotional intelligence had positive effects on the burnout of nurses [12,13]. Since the effects of emotional intelligence on burnout are inconsistent, further research on this issue is necessary to compare various occupations in different types of healthcare organizations. 

Despite the usefulness of the results, the study has some limitations. First, an online self-reported questionnaire could create errors in data collection. We attempted to manage duplicates in responses through cross-checking the cell phone number and sociodemographic characteristics of each respondent. Second, the analysis was performed using survey responses from four general hospitals in a metropolitan city. This number may limit a generalization of the study findings. Lastly, the cross-sectional design would not confirm the causality. Longitudinal studies to assess the direction of the relationship between emotional intelligence and burnout is recommended. 

## 5. Conclusions

This study contributes to understanding the relationships between job stress and burnout, and the emotional intelligence and burnout in general hospital administrative staff. Under the JD-R model, a demanding workload would cause job stress which, in turn, leads to burnout. The study implies that factors other than work-related charteristics need to be considered in order to decrease burnout levels in administrative staff. Emotional intelligence, which is a personal dimension, is found to be a direct influencing factor, rather than moderating effect, on burnout. Based on the aforementioned findings, it is, therefore, believed that development and implementation of programs that could decrease job stress at the organizational level and increase emotional intelligence abilities at the individual level would be helpful in alleviating burnout levels in general hospital administrative staff.

## Figures and Tables

**Table 1:** Differences in burnout by general characteristics (*N* = 191).

Variables	Categories	M ± SD	*n* (%)		Burnout	
					M ± SD	t/F (*p*)
Gender	Male		74	(38.74)	2.78 ± 0.82	−1.90 * (0.06)
	Female		117	(61.26)	2.99 ± 0.73	
Age (year)		35.72 ± 8.59				
	20–29		55	(28.80)	3.06 ± 0.85	65.88 (0.34)
	30–39		73	(38.22)	2.89 ± 0.74	
	≥40		63	(32.98)	2.79 ± 0.73	
Marital status	Single		90	(47.12)	2.99 ± 0.88	−1.30 (0.19)
	Married		101	(52.88)	2.84 ± 0.70	
Education level	Community college		24	(12.27)	2.82 ± 0.72	62.14 (0.47)
	University		128	(67.02)	2.95 ± 0.78	
	Graduate school		39	(20.42)	2.81 ± 0.79	
Religion	Yes		79	(41.36)	2.81 ± 0.71	1.42 (0.15)
	No		112	(58.64)	2.98 ± 0.80	
Length of career (year)		10.08 ± 8.17				
	<3		39	(20.42)	2.93 ± 0.85	86.07 (0.68)
	3~<7		47	(24.61)	3.00 ± 0.86	
	7~<15		51	(26.70)	2.83 ± 0.66	
	15≥		54	(28.27)	2.89 ± 0.75	
Work position	Staff		155	(81.15)	2.93 ± 0.79	0.92 (0.36)
	≥Middle manager		36	(18.85)	2.80 ± 0.65	
Work department	patient contact		75	(39.27)	2.95 ± 0.75	−0.65 (0.51)
	patient non-contact		116	(60.73)	2.88 ± 0.79	

Abbreviations: M, mean; SD, standard deviation. * *p* < 0.10.

**Table 2:** Properties of job stress, emotional intelligence, and burnout questionnaires.

Variables	Number of Items	Range Observed	Mean	SD
Job stress	24	17.46~079.76	47.51	12.11
Emotional Intelligence	14	3.36~6.93	5.28	0.64
Burnout	9	1.11~4.89	2.91	0.77

Abbreviation: SD, standard deviation.

**Table 3:** Correlation matrix for job stress, emotional intelligence, and burnout.

Variables	Job Stress	Emotional Intelligence	Burnout
Job stress	1.0		
Emotional intelligence	−0.27 ***	1.0	
Burnout	0.56 ***	−0.26 ***	1.0

*** *p* < 0.001.

**Table 4:** Influencing factors on burnout (*N* = 191).

Variables	Model 1		Model 2		Model 3	
	**β**	**(*se*)**	**β**	**(*se*)**	**β**	**(*se*)**
Constant	2.9	(0.21)	0.95	(0.59)	2.23	(0.51)
Gender (male = ref.)	0.18	(0.12)	0.06	(0.09)	0.06	(0.09)
Age (20s = ref.)						
30–39	−0.11	(0.16)	−0.19	(0.13)	−0.20	(0.13)
≥40	−0.17	(0.19)	−0.43 **	(0.16)	−0.44 **	(0.16)
Marital Status (married = ref.)						
Single	0.01	(0.15)	−0.12	(0.12)	−0.12	(0.13)
Religion (no = ref.)						
Yes	−0.12	(0.12)	−0.09	(0.09)	−0.09	(0.09)
Work department (patient non-contact = ref.)						
Patient contact	0.09	(0.12)	−0.07	(0.09)	−0.07	(0.09)
Job Stress (JS)			0.04 ***	(0.12)	0.04 ***	(0.12)
Emotional Intelligence (EI)			−0.14 *	(0.07)	−0.14 *	(0.08)
JS X EI					0.00	(0.006)
R^2^	0.038		0.377		0.378	
Adj. R^2^	0.008		0.349		0.347	
F	1.24		13.74 ***		12.24 ***	

Abbreviation: *se*, standard error. * *p* < 0.10; ** *p* < 0.05; *** *p* < 0.001.

## Data Availability

The data presented in this study are available on request from the corresponding author. The data are not publicly available due to data restriction policies.
